# Efficacy of radiotherapy in combined treatment of hepatocellular carcinoma patients with portal vein tumor thrombus: a real-world study

**DOI:** 10.1186/s12893-024-02334-1

**Published:** 2024-02-14

**Authors:** Ying Xiao, Keren Li, Ying Zhao, Shizhong Yang, Jun Yan, Canhong Xiang, Jianping Zeng, Qian Lu, Chen Zhang, Gong Li, Guangxin Li, Jiahong Dong

**Affiliations:** 1 Department of Pathology, Beijing Tsinghua Changgung Hospital, School of Clinical Medicine, Tsinghua University, Beijing, China; 2 Hepatopancereatobiliary Center, Beijing Tsinghua Changgung Hospital, School of Clinical Medicine, Tsinghua University, Beijing, China; 3 Department of Radiation Oncology, Beijing Tsinghua Changgung Hospital, School of Clinical Medicine, Tsinghua University, 168 Litang Road, Changping District, Beijing, China; 4 Imaging department, Beijing Tsinghua Changgung Hospital, School of Clinical Medicine, Tsinghua University, Beijing, China; 5 Research Unit of Precision Hepatobiliary Surgery Paradigm, Chinese Academy of Medical Sciences, Beijing, China

**Keywords:** Hepatocellular carcinoma, Portal vein tumor thrombosis, Radiotherapy, RACIB

## Abstract

**Background and aims:**

Hepatocellular carcinoma (HCC) with portal vein tumor thrombus (PVTT) has an extremely poor prognosis. A previous study proved that low-dose radiotherapy (RT) could prolong the prognosis of HCC patients with PVTT. This study aims to explore the sensitivity of PVTT to RT treatment.

**Methods:**

Patients were selected based on imaging diagnosis of HCC accompanied by PVTT and received combined treatment of radiotherapy, antiangiogenic drugs and immune checkpoint inhibitors, followed by hepatectomy or liver transplantation from January 2019 to August 2022. The efficacy was evaluated by Response Evaluation Criteria in Solid Tumors (RECIST) guidelines and pathological assessment. The sensitivity of tumor cells to the treatment was compared between the primary tumor (PT)and PVTT by analyzing their residual tumor and pathologic complete remission (PCR) incidence.

**Results:**

Data from 14 patients were collected in the study. After combined treatment, the size of PVTT decreased more significantly than that of the primary tumor in the imaging study (*p* < 0.05). The residual cancer was significantly more restrictive than that of primary tumor in paired patients based on pathological measurement (*p* = 0.008). The PCR incidence of the primary tumor (21.42%) was significantly lower (*p* = 0.008) than that of PVTT in the pathologic study (78.57%).

**Conclusion:**

PVTT is more sensitive to radiotherapy treatment than the primary tumor in patients with HCC. This combination therapy might be an effective option as a downstaging therapy for patients with HCC with PVTT.

**Supplementary Information:**

The online version contains supplementary material available at 10.1186/s12893-024-02334-1.

## Introduction

Hepatocellular carcinoma (HCC) is the main type of liver cancer, which was the seventh most common cancer and the fifth leading cause of cancer deaths according to 2022 global cancer statistics [1]. Due to its lack of symptoms at an early disease stage, 44-62.2% of HCC patients have portal vein tumor thrombus (PVTT) at first diagnosis [2], resulting in the extremely poor prognosis of HCC with PVTT. According to the Barcelona Clinic Liver Cancer Staging (BCLC) staging system, HCC patients with PVTT are at the advanced stage (stage C) [3]. The median survival time of these patients without any treatment is only 2.7 months [4]. The LEAP-002 study showed that the median OS was 17.8 months in advanced HCC patients with PVTT but excluding patients with tumor thrombosis of the main trunk of the portal vein (Vp4) [5]. Various treatment modalities such as surgical resection, three-dimensional radiotherapy (3D-RT), stereotactic body radiotherapy (SBRT), transarterial chemoembolization (TACE), hepatic arterial infusion chemotherapy (HAIC), multikinase inhibitors and immunotherapy, and even hepatectomy, have been used to treat advanced HCC with PVTT, but the optimal treatment strategy remains controversial. As reported, surgical resection improves the survival of patients with PVTT to 6.2–64 month [2, 6]. However, HCC patients with PVTT of type III to IV (Cheng’s PVTT classification system) have limited benefit from surgical resection alone [7]. Multikinase inhibitors such as sorafenib and lenvatinib are recommended as first-line treatments but can only extend the median survival time to 12.3–13.6 month [8]. The IMbrave150 study showed that the median OS was only 7.6 months in patients with Vp4 PVTT treated with sorafenib [9]. Consequently, it is critical to explore an effective treatment for these patients.

Radiotherapy (RT) is an effective treatment various cancer. Its safety and efficacy for treating HCC patients with PVTT have been verified [10, 11]. Recent studies have indicated that RT can enhance antitumor immunity when combined with immune checkpoint inhibitors [12]. RT plus anti-PD1 showed significantly promising efficacy in patients with advanced HCC [13]. RT alone or combined with other therapies has the potential for tumor downstaging, creating surgical opportunities for more advanced patients [14–16]. The LEAP-002 study of lenvatinib plus pembrolizumab treatment showed promising objective response rates (ORRs), including in some patients with PVTT distal to the second-order branches of the portal vein (Vp1), in the second-order branches (Vp2) and in the first-order branches (Vp3) [5]. This suggests that RT combined with antiangiogenesis and immune checkpoint blockade inhibition (RACIB) may potentially play a critical role in improving the efficacy of treatment. A randomized, open-label, multicenter controlled clinical study showed that 17 patients (20.7%) had partial remission in the neoadjuvant RT group, proving that neoadjuvant RT significantly reduced HCC-related mortality and HCC recurrence rates compared with surgery alone. However, we found in this study that the PVTT apparently shrank after low-dose (18 Gy/6 F) radiation [17]. This phenomenon suggested that PVTT may be more sensitive to RT.

Thus, we designed this study to assess the sensitivities of PVTTs to RT by evaluating postoperative imaging and pathological tumor response, and comparing to the paired primary lesions.

## Materials and methods

### Study design and patients

The aim of this retrospective study was to investigate the RT sensitivity of PVTTs and primary lesions in patients who underwent preoperative RT combined with angiogenesis and immune checkpoint blockade inhibitors at Beijing Tsinghua Changgung Hospital. The clinical and pathological data of the patients were collected. The study was approved by the institutional review board of Beijing Tsinghua Changgung Hospital and exempt from ethics approval. HCC patients with PVTT who had undergone hepatectomy or liver transplantation from January 2019 to August 2022 were retrospectively identified. The eligibility criteria of the patients were as follows: (a) age between 20 and 80 years; (b) HCC diagnosed by imaging or pathology according to the EASL Clinical Practice Guidelines; (c) HCC with Vp3 or Vp4 PVTT; (d) no distant metastasis; and (e) external beam RT combined with lenvatinib and the PD-1 inhibitor sintilimab treatment for PVTT.

The exclusion criteria were as follows: (a) patients who had positive nodal or extrahepatic metastases; (b) Eastern Cooperative Oncology Group Performance Status 2 or greater; (c) Child‒Pugh classification of class C disease; and (d) patients who did not undergo surgery after RT.

### Selection of subjects

Patients were diagnosed either by pathologic confirmation of HCC or by typical radiological hallmarks of HCC using two dynamic imaging studies, namely, computed tomography (CT) with contrast enhancement, magnetic resonance imaging (MRI) or hepatic angiography. The details of the selection of subjects for pathology and imaging are shown in the supplementary material.

### Combined therapies

Lenvatinib was started on the first day of RT (daily dose determined according to body weight, 8 mg for bodyweight < 60 kg and 12 mg for bodyweight ≥ 60 kg) and discontinued 7 days before surgery.

The PD-1 inhibitor (sintilimab) was also started on the first day of RT, with a fixed dose of 200 mg every three weeks for three cycles.

The indications of preoperative RT and the details (dose and routine) can been found in in the supplementary material.

Surgery was performed 6 to 8 weeks after RT.

### Tumor response and toxicity evaluation

#### Serum tumor markers

The preoperative serum levels of a-fetoprotein (AFP), protein induced by the absence of vitamin K or antagonist-II (PIVKA-II), alanine aminotransferase (ALT), aspartate aminotransferase (AST), gamma-glutamyl transferase (GGT), alkaline phosphatase (ALP), bilirubin and creatinine were recorded as were the hemoglobin level and counts of neutrophils and platelets. They were compared before and after RACIB.

### Radiological evaluation

The treatment response after RT was evaluated using CT or MRI every 8–12 weeks. Abdominal CT scans were taken before and after RACIB. The tumor response of both the PT and PVTT was evaluated by Response Evaluation Criteria in Solid Tumors (RECIST) guidelines using abdominal CT scans. Complete response (CR), partial response (PR), stable disease (SD), or progressive disease (PD) were determined by using multiphasic contrast-enhanced CT and MRI.

### Pathological evaluation

The median period from the final day of RT to surgery was 99 (range 3–761) days. The pathological responses of the irradiated PVTT and PT specimens were evaluated. The surgical specimen of the liver was sectioned in parallel at 0.5 cm intervals. The number, size and location of the residual tumor or fibrotic tumor bed were determined and recorded. If the tumor was less than 5 cm, all tumor regions were obtained. If the tumor was larger than 5 cm, two representative surfaces were obtained (if no microscopic tumor cells were observed in the initial sections, all residual tumor samples were obtained). All PVTTs were sampled for evaluation. The proportion, distribution, and differentiation of residual tumor cells in both the tumor bed and PVTT were assessed microscopically. CR was defined as no living tumor cells in either the PT bed or PVTT. The areas were replaced by tumor necrosis and fibroelastic connective tissues with foamy macrophages and chronic inflammatory cells.

### Statistical analysis

Data were analyzed by SPSS software (version 27.0; SPSS Inc., Chicago, IL, USA). Data on the residual cancer burden from paired patients were analyzed by paired t tests. Data on CR incidence were analyzed by chi-square tests. IHC staining data for paired HCC samples were analyzed by chi-square tests. A two-sided *p* value < 0.05 was considered statistically significant.

## Results

### Patient characteristics

A total of 71 patients with HCC with PVTT underwent hepatectomy or liver transplantation. 57 were excluded because they did not receive preoperative RT. Finally, 14 patients were enrolled in this study. They had a mean age of 56.07 ± 10.27 years. Two of them were women and the remaining 12 were men. The mean size of primary HCC in the liver parenchyma was 7.84 ± 3.99 cm. Six patients had multiple liver tumors. The corresponding numbers of types II, III and IV were 8, 5 and 1 respectively, according to the Cheng’s PVTT classification system. And the corresponding numbers of type VP2, VP3 and VP4 were 1, 6 and 7 according to the Japanese PVTT classification system. The baseline characteristics of the patients are listed in Table 1.

**Table 1:** Baseline characteristics of HCC patients with PVTT

Case	Age (year)	sex	Primary tumor size (cm)	Primary tumor number	Subtype of PVTT (Japan)	Subtype of PVTT (Chen)
1	59	Female	2.9	single	VP4	3B
2	53	Male	14.5	single	VP4	4
3	43	Male	4.9	multiple	VP2	2
4	70	Male	4.3	single	VP4	3a
5	51	Male	2	multiple	VP3	2
6	52	Male	10	multiple	VP3	3
7	48	Male	9.4	multiple	VP4	2
8	68	Male	8.3	single	VP4	2
9	53	Male	12	single	VP3	2
10	46	Male	2.2	single	VP3	2
11	45	Male	9	single	VP3	3
12	59	Male	12	multiple	VP4	2
13	78	Female	7.6	multiple	VP3	2
14	60	Male	10.7	single	VP4	3

In this study, all patients received RT followed by lenvatinib combined with the immune checkpoint blockade inhibitor sintilimab. The RT doses for primary foci and PVTTs were consistent for each individual pair, but varied between independent individuals. Due to differences in patients’ tolerance to RT, 10 of them received low-dose RT (less than 50 Gy in total) and the other 4 received higher-dose RT. The dosage of oral medicine was the same for every patient. The details of the treatment information are listed in Table 2. All patients met the criteria for surgical excision after RACIB. Nine of them underwent the liver transplantation, and the other 4 patients underwent the hepatectomy. The surgical criteria of the patients can be seen in the supplementary material.

**Table 2:** Treatment information of HCC patients with PVTT

Case	RT dose	RT dose level	PTV(CC)	Normal liver PTV (CC)	PT RECIST	PVTT RECIST	Operation	PT cancer burden		PVTT cancer burden
1	3 Gy×10f	low	91.4	844.4	SD	SD	transplantation	70%		0%
2	2 Gy×20f	low	1858.2	992	SD	SD	transplantation	10%		0%
3	4.5 Gy×10f	low	495	1066.2	SD	SD	transplantation	70%		60%
4	5 Gy×8f	low	360	1691.9	SD	SD	transplantation	20%		10%
5	2 Gy×23f	low	661.3	971.3	PR	PR	transplantation	90%		0%
6	3 Gy×10f	low	1700	1026.6	PR	SD	right	50%		50%
7	2 Gy×20f	low	955	1433.5	SD	SD	transplantation	60%		0%
8	2 Gy×25f	high	72	905.9	PR	PR	right	70%	0%	
9	2 Gy×18f + 2 Gy×7f	high	988.9	1665.4	PR	PR	right	10%	0%	
10	2 Gy×20f	low	474.5	953.5	PR	PR	transplantation	0%	0%	
11	3 Gy×20f	high	859.2	1133	PR	PR	transplantation	0%	0%	
12	3 Gy×20f	high	1026.8	1338	PR	PR	left	20%	0%	
13	2 Gy×20f	low	438.1	10.575	PR	PR	right	0%	0%	
14	3 Gy×10f	low	157.759	2128.38	SD	SD	transplantation	20%	0%	

PT: primary tumor; PVTT: portal vein tumor thrombus; RECIST: response evaluation criteria in solid tumors; RT: radiotherapy;

### Comparison of tumor response and toxicity before and after RT treatment

**Fig. 1:**
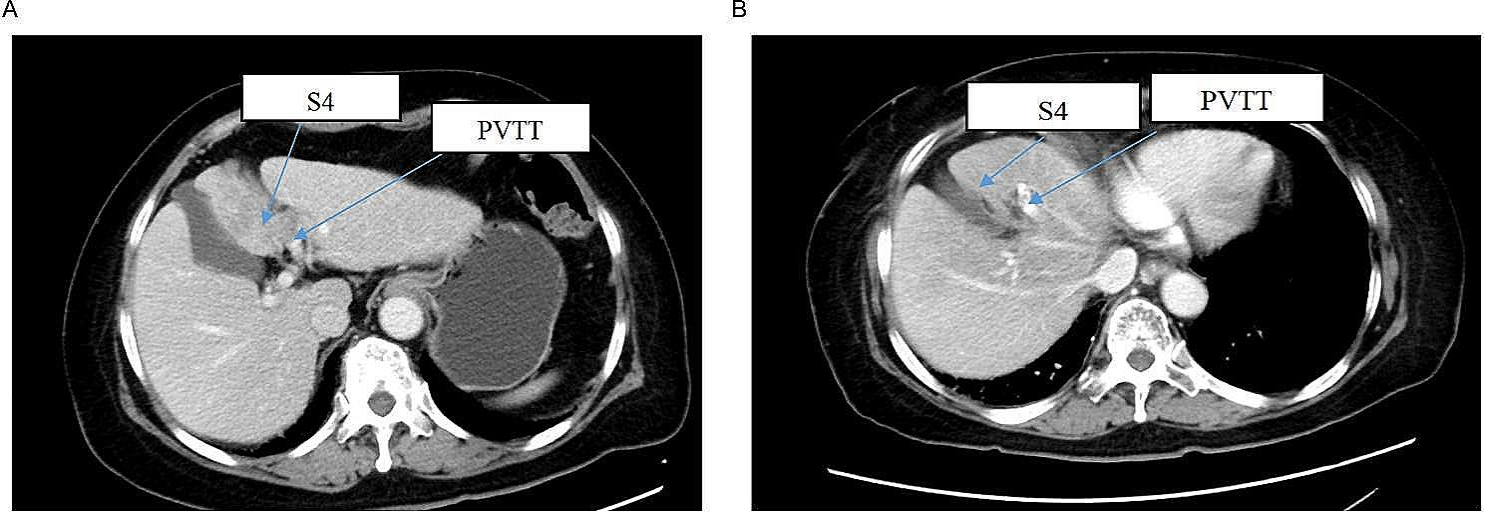
Representative sequential MRI images for one patient. (**A**) The images show the primary tumor and PVTT before RT treatment. (**B**) The images show the primary tumor and PVTT after RT treatment MRI: magnetic resonance imaging; PT: primary tumor; PVTT: portal vein tumor

The sizes of the PTs and PVTTs were both decreased significantly after RT-based treatment (Fig. 1). The pretreatment mean size of the PTs was 7.84 ± 3.99 cm and the posttreatment mean size was 6.28 ± 3.96 (*p* = 0.034). The PVTT size was decreased from 1.99 ± 1.80 cm to 0.14 ± 0.33 cm (*p* = 0.002). After RACIB, 6 (42.86%) patients had SD and 8 (57.14%) had PR for PT according to the RECIST guidelines on abdominal CT scans. Meanwhile, 7 (50%) patients had SD and 7 (50%) had PR for PVTT (Table 2). However, there was no difference in AFP and PIVKA-II levels before and after RT-based treatment (Table 3).

**Table 3:** Efficacy biomarkers before and after RT-based preoperative treatment

Parameter	Before	After	*P*
Primary tumor size (cm)	7.84 ± 3.99	6.28 ± 3.96	0.034
PVTT size (cm)	1.99 ± 1.80	0.14 ± 0.33	0.002
AFP(log_10_)	2.48 ± 1.59	1.73 ± 1.51	0.054
PIVKA-II(log_10_)	3.26 ± 1.09	2.53 ± 1.16	0.075

*p* < 0.05 was considered statistically significant

AFP: a-fetoprotein; PIVKA-II: protein induced by the absence of vitamin K or antagonist-II; RT: radiotherapy;

The toxicity of the RACIB is listed in Table 4. Two patients had grade III hepatic toxicity, one had a decrease in grade III neutrophils decreased, and one had a decrease in grade IV platelets decreased. They all returned to normal after treatment. No gastrointestinal toxicity was observed. The Child‒Pugh score increased by no more than 2 points.

**Table 4:** The toxicity of RT-based preoperative treatment

Parameter	Number of the patients	%
Liver enzyme levels increased		
0	3	21.43
I	3	21.43
II	6	42.86
III	2	14.29
Bilirubin increased		
0	8	57.14
I	5	35.71
II	1	7.14
Creatinine increased		
0	13	92.86
I	1	7.14
Anemia		
0	10	71.43
I	2	14.29
II	2	14.29
Neutrophils decreased		
0	11	78.57
II	2	14.29
III	1	7.14
Platelets decreased		
0	4	28.57
I	9	64.29
IV	1	7.14

### Comparation of tumor response between PTs and PVTTs in paired arrays

**Fig. 2:**
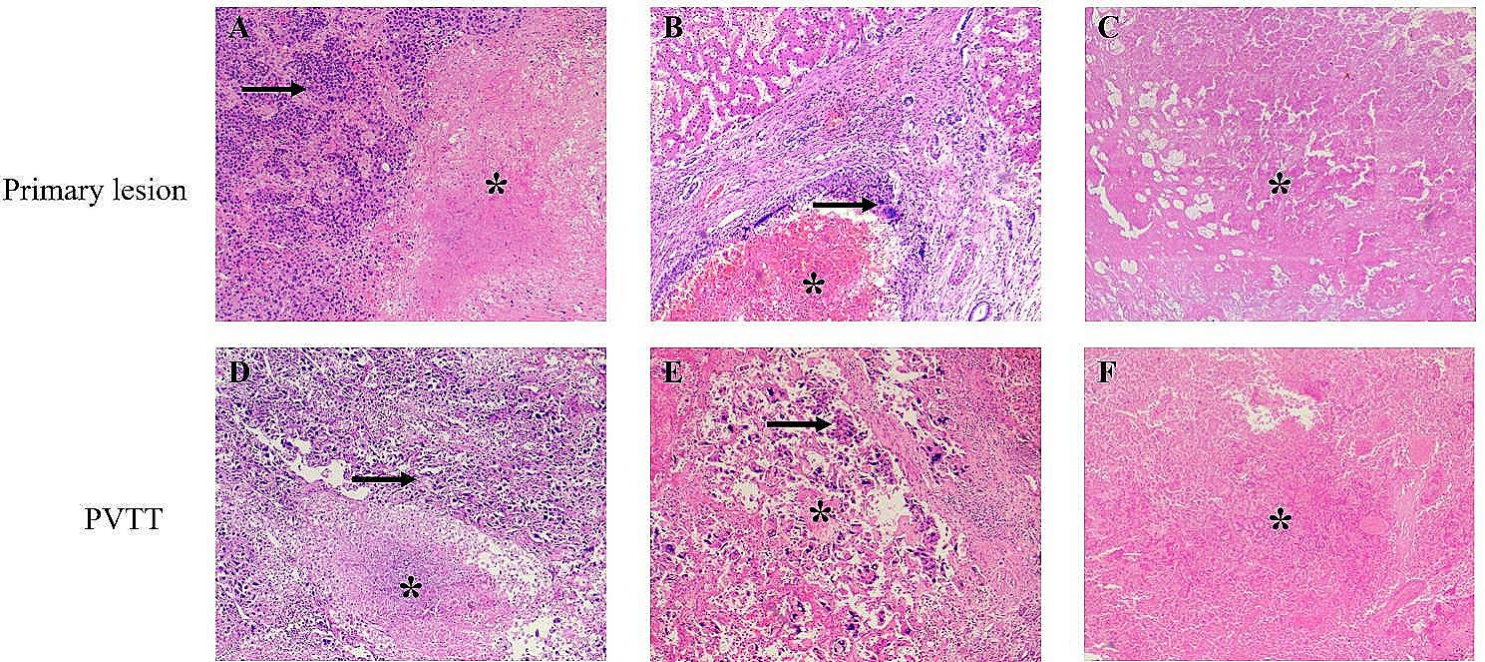
Microscopic findings of primary lesions and PVTT with different therapeutic effect. (**A**)-(**C**) Tumor necrosis and residual tumor cells of the primary lesions in different patients. (**D**)-(**F**) Tumor necrosis and residual tumor cells of PVTT in different patients. *: Tumor necrosis. →: residual tumor cells PVTT: portal vein tumor

Microscopic observation revealed that tumor necrosis was unevenly distributed in both the PT and PVTT and lacked inflammatory cell infiltrate. The necrotic focus was more concentrated in the center, and the residual tumor cells were in the periphery of the tumor bed (Fig. 2A-B, D-E). Nuclei in the residual tumor cells exhibited degeneration with chromatin clumping or smudging. Patients who were sensitive to RACIB exhibited complete tumor necrosis on pathological evaluation (Fig. 2C, F).

**Fig. 3:**
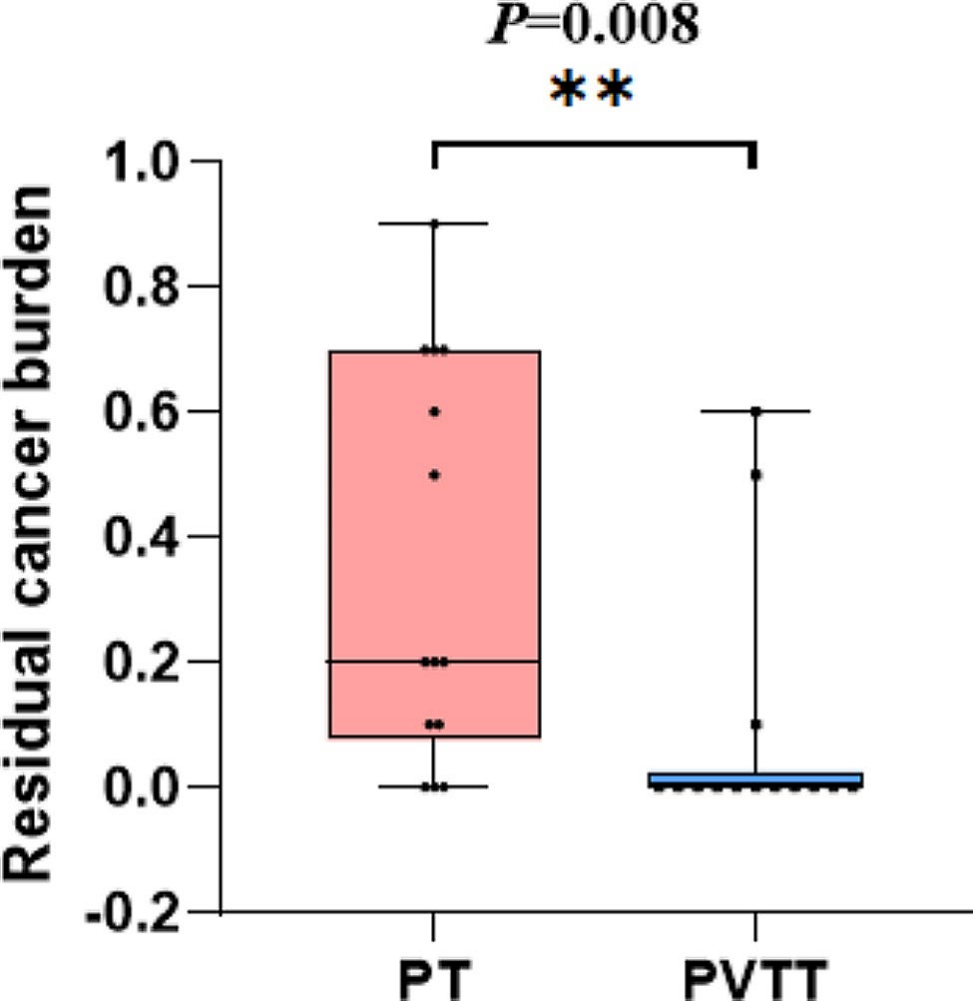
The comparison of residual cancer burden between PT and PVTT. *p < 0.05, **p < 0.01, ***p < 0.001 PT: primary tumor; PVTT: portal vein tumor

Residual cancer burden and pathological complete response (PCR) are important markers for estimating the downstaging of HCC. We compared them between PTs and PVTTs in paired arrays to analyze their differences in sensitivity to RACIB. The residual cancer burden of the PTs (35.00 ± 31.81) was significantly higher (*p* = 0.008) than that in PVTTs (8.57 ± 19.95) (Fig. 3). The incidence of PCR of PTs (21.42%) was significantly lower than that of PVTTs (78.57%) (Table 5).

**Table 5:** The difference of pCR-incidence between PT and PVTT

Parameter	PT(*n* = 14)	PVTT(*n* = 14)	*P*
PCR	3(21.42%)	11(78.57%)	**0.008**
Non-PCR	11(78.57%)	3(21.42%)	

Chi-square test was performed for statistical analysis. *p* < 0.05 was considered statistically significant

PCR: pathologic complete remission; PT: primary tumor; PVTT: portal vein tumor thrombus;

## Discussion

Hepatocellular carcinoma (HCC) with portal vein tumor thrombus (PVTT) is often regarded as advanced stage disease according to the BCLC staging classification [3]. However, apart from PVTT, extrahepatic metastasis including to hilar lymph nodes, retroperitoneal lymph nodes and distant organs is often defined as BCLC C stage as well [18, 19]. The biological behavior of advanced HCC accompanied by only PVTT is actually quite different from the biological behavior of HCC with extrahepatic metastasis. Therefore, the China Liver Cancer (CNLC) staging system refers to the stage of HCC with only PVTT as stage IIIa, while the stage with extrahepatic metastasis is referred to as stage IIIb [20]. For patients with HCC accompanied by PVTT, a number of researchers have tried different local treatments, such as TACE, HAIC, Y90 combined with systematic therapy and even resection, and obtained promising results [21–23].

A systematic review demonstrated that the median overall survival of HCC patients with PVTT treated with surgical resections ranged from 8.2 to 30 months.While the median overall survival of HCC patients with PVTT received non-surgical treatments ranged from 7 to 13.3 months [24].

The recent IMbrave150 study showed that antiangiogenesis combined with ICB could prolong the median overall survival of advanced HCC patients to 22 months, among which that of the median survival of patients with PVTT Vp4 was 7.6 months, much higher than that of control group [9]. This study revealed that antiangiogenesis combined with ICB was an effective treatment for PVTT. Keynote-524 also showed that the efficiency of antiangiogenesis combined with ICB was 42.3-48%, which could rapidly decrease the tumor volume of liver cancer rapidly [25]. This study also demonstrated that HCC with PVTT can change from an unresectable to a resectable status, and the transformation efficiency was 30%.

A previous study showed that RT can induce immune responses such as activation and recruitment of antitumor immune subsets (CD4+, CD8 + T cells, cytotoxic NK cells, and CD8 + CD56 + natural killer T (NKT) cells) to the TME [26–28]. Furthermore, clinical studies have reported upregulated expression of PD-1 and PD-L1 by CD8 + T cells and tumor cells, respectively, post-RT [29, 30]. Therefore, the combination of RT and immunotherapy could produce synergistic antitumor immunity for durable disease control.

However, previous study showed that it was difficult to evaluate the efficiency RT for treating PVTT because it was unmeasurable by imaging. A Korean study showed that there was no difference in overall survival between only PVTT with RT and both PVTT and PT with RT in advanced HCC patients [31]. In fact, liver cancer went down from BCLC C to BCLC A once PVTT necrosis was complete. Japanese researchers found that PCR could be achieved in 83.3% of PVTTs treated with 2 weeks of RT (30–36 Gy) before resection [32]. In general, PTs were treated with at least 60 Gy, and even 100 Gy was associated with PCR. This indicated that PVTT was sensitive to RT. It could improve HCC patients’ liver function and prolong their survival once the PVTT decreased or vanished, which improved surgery opportunities for more patients with advanced HCC.

In this study, the PCR incidence of PVTT was significantly higher than that of the PT (78.57% vs.21.42%) and the ratio of SD of PVTT was higher than that of the PT (50% vs.42.86%) after RACIB. Additionally, PR in both the PT and PVTT and PCR in the PVTT were achieved after high-dose RT (4 patients). None of the patients experienced greater than grade II toxicity. Three patients who received low-dose RT had greater than grade III toxicity, which may be because the wide field of RT was caused by the large volume of PTs. However, they all recovered after symptomatic treatment, indicating that their side effects were manageable. Our study proved that RACIB might be an effective and safe treatment for HCC patients with PVTT.

In addition to these results, there are some limitations in this study. First, it was limited by its retrospective design. Moreover, due to the limitations of the follow-up sample size, there was no difference in AFP and PIVKA-II levels before and after RT-based treatment. Subsequent verification with larger samples and prospective studies are required to increase the level of evidence. Furthermore, a uniform RT dose is needed in future studies. However, this treatment is worth promoting and administering to patients with HCC with PVTT.

## Conclusion

In summary, our present work indicated that PVTTs are more sensitive than PTs to RT in patients with HCC. RT combined with antiangiogenesis and immune checkpoint blockade inhibition (RACIB) creates surgical opportunities for advanced HCC patients and might be a promising treatment for downstaging HCC patients with PVTT in the future.

### Electronic supplementary material

Below is the link to the electronic supplementary material.

Supplementary Material 1

## Data Availability

No datasets were generated or analysed during the current study.

## Abbreviations

BCLC: Barcelona Clinic Liver Cancer
CR: Complete Response
HCC: Hepatocellular carcinoma
OS: Overall survival
PCR: Pathologic Complete Remission
PD: Progressive Disease
PR: Partial Response
PT: Primary tumor
PVTT: Portal Vein Tumor Thrombus
RACIB: RT combined with anti-angiogenesis and immune checkpoint blockade inhibition. RECIST:Response Evaluation Criteria in Solid Tumors
RT: Radiotherapy
SD: Stable Disease
