# Supplementary material for: Efficacy of radiotherapy in combined treatment of hepatocellular carcinoma patients with portal vein tumor thrombus: a real-world study
Source: BMC Surg. 2024 Feb 14;24:54. doi: 10.1186/s12893-024-02334-1 (PMC10865509; doi:10.1186/s12893-024-02334-1)
Supplement: Supplementary file 1 — Supplementary Material 1 [file 12893_2024_2334_MOESM1_ESM.docx]

1.**Selection of subjects**

1.1 Diagnosis criteria of Pathology

Pathological diagnosis of HCC should be based on the International Consensus recommendations using the Pathological diagnosis of HCC should be based on the

International Consensus recommendations using the required histological and immunohistological analyses. Non-invasive criteria can only be applied to cirrhotic patients for nodule(s) ≥1 cm, in light of the high pre-test probability and are based on imaging techniques obtained by multiphasic CT, dynamic contrast-enhanced.

1.2 Diagnosis criteria of Imaging

1.2.1 Magnetic Resonance Imaging (MRI)

MRI according to EASL Clinical Practice Guidelines: Management 2018）Diagnosis and Classification of PVTT include solidlesions within the portal vein in all the phases of intravenous enhanced three-phase computed tomography, especially with an enhancement of contrast in the arterial phase and washout in the portal venous phase of the procedure in accordance with Guidelines for Diagnosis and Treatment of Hepatocellular Carcinoma with Portal Vein Tumor Thrombus in China (2021 Edition)

1.2.2 Computed Tomography (CT) with contrast enhancement

CT scan（Discovery590 RT,GE,USA）was performed with the patient in the supine position with chest-abdominal thermoplastic mask immobilization to reduce uncertainty and restrain organ motion caused by abdominal breathing. The Monaco 5.11 treatment planning systems (Elekat, Sweden) was used optimize target and normal structure delineation. External RT with volumetric modulated arc therapy (VMAT) treatment plans targeting the PVTT and primary tumor. The gross tumor volume (GTV) of PVTT was defined as the tumor volume that was shown as a filling defect in the portal venous phase of the CT scan. The planning target volume (PTV) of PVTT was expanded to include the margin of 5 mm in the transaxial direction and abdominal-dorsal direction and 10 mm in the cranio-caudal direction. The GTV of primary tumor was defined as the liver tumor volume that was enhanced in the arterial phase of CT scan. The clinical tumor volume (CTV) of primary tumor was generated by adding 5 mm to the GTV in all direction. The PTV of primary tumor was expanded to include a 5- to 10-mm margin from the CTV to compensate for internal physiologic movements and variations in size, shape, and position of the CTV. The total dose to the PTV was 30-60 Gy, with a fractional size of 2.0-3.0 Gy, using 6-MV x-rays with a linear accelerator (Synergy; Elekta, Sweden) at 5 fractions per week. Mean dose to the normal liver (volume of total liver minus GTV) was limited to ≤23 Gy, The maximum allowable point dose to the duodenum and stomach should be less than 54 Gy. The maximum allowable point dose to colon was less than 55 Gy. The maximum point dose of cord was less than 45 Gy. The kidney volume receiving a dose ≥20 Gy (V20) was <20%.

**2.Surgical criteria**

2.1 criteria of hepatectomy

A) The liver remnant volume is lager than the 40% of the original volume

B) The liver function meet Child-Pugh class A.

2.2 criteria of liver transplantation

The cirrhosis is too serious to undergo liver resection.

1. **Criteria of Radiotherapy (RT)**
   1. indications of preoperative RT
2. Aged 20-80 years.
3. The primary tumor was resectable: the remaining liver vascular structure was intact and the liver volume was sufficient, which was in line with the decision-making system for safe hepatectomy; or the patient was suitable for liver transplantation.
4. ECOG performance status 0-1.
5. Child-Pugh score ≤ 7.
6. HBV DNA <500 IU/ml and had been receiving conventional antiviral therapy for HBV antigen-positive patients.
7. For normal function of major organs (the following criteria should be met: Adequate bone marrow function, defined as:
8. Absolute neutrophil count (ANC ≥1.5 x 10^9 /L);
9. Hemoglobin (Hb ≥8.5 g/dL) ;
10. Platelet (PLT ≥ 75 x 10^9/L);
11. Adequate liver function, defined as: Albumin ≥ 2.8 g/dL, Bilirubin ≤3.0 mg/dL，Aspartate aminotransferase (AST), alkaline phosphatase (ALP) and alanine aminotransferase (ALT) were ≤ 5 times the upper limit of normal (ULN);
12. Adequate coagulation function, defined as: International Normalized Ratio (INR) of 2.3 or less;
13. Adequate renal function, defined as: creatinine clearance ＞ 40 mL/min, calculated according to the Cockcroft and Gault formula;
14. Adequate pancreatic function, defined as: amylase and lipase ≤ 1.5 times ULN).
15. No pregnancy or planned pregnancy.
    1. details of RT

CT scan（Discovery590 RT,GE,USA）was performed with the patient in the supine position with chest-abdominal thermoplastic mask immobilization to reduce uncertainty and restrain organ motion caused by abdominal breathing. The Monaco 5.11 treatment planning systems (Elekat, Sweden) was used optimize target and normal structure delineation. External RT with volumetric modulated arc therapy (VMAT) treatment plans targeting the PVTT and primary tumor. The gross tumor volume (GTV) of PVTT was defined as the tumor volume that was shown as a filling defect in the portal venous phase of the CT scan. The planning target volume (PTV) of PVTT was expanded to include the margin of 5 mm in the transaxial direction and abdominal-dorsal direction and 10 mm in the cranio-caudal direction. The GTV of primary tumor was defined as the liver tumor volume that was enhanced in the arterial phase of CT scan. The clinical tumor volume (CTV) of primary tumor was generated by adding 5 mm to the GTV in all direction. The PTV of primary tumor was expanded to include a 5- to 10-mm margin from the CTV to compensate for internal physiologic movements and variations in size, shape, and position of the CTV. The total dose to the PTV was 30-60 Gy, with a fractional size of 2.0-3.0 Gy in 12 patients and another 2 patients were given 40-45Gy to PTV with a fractional size of 4.5-5.0Gy using 6-MV x-rays with a linear accelerator (Synergy; Elekta, Sweden) at 5 fractions per week. Mean dose to the normal liver (volume of total liver minus GTV) was limited to ≤23 Gy, The maximum allowable point dose to the duodenum and stomach should be less than 54 Gy. The maximum allowable point dose to colon was less than 55 Gy. The maximum point dose of cord was less than 45 Gy. The kidney volume receiving a dose ≥20 Gy (V20) was <20%.
